# Attenuation of Collagen-Induced Arthritis in Mice by Salmon Proteoglycan

**DOI:** 10.1155/2014/406453

**Published:** 2014-05-22

**Authors:** Sayuri Yoshimura, Krisana Asano, Akio Nakane

**Affiliations:** Department of Microbiology and Immunology, Hirosaki University Graduate School of Medicine, 5 Zaifu-cho, Hirosaki, Aomori 036-8562, Japan

## Abstract

Rheumatoid arthritis (RA) is a serious autoimmune disease caused by chronic inflammation of connective tissues. The basic principle of RA treatment is aimed to reduce joint inflammation. Our previous studies demonstrated that salmon cartilage proteoglycan (PG) suppresses excess inflammation in different mouse inflammatory diseases. In this study, we investigated the prophylactic effect of PG on the progression of RA using an experimental mouse model, collagen-induced arthritis (CIA). Clinical and histological severity of CIA was attenuated by daily oral administration of PG. In the joints of PG-administered mice, infiltration of macrophages and neutrophils and also osteoclast accumulation were limited. In comparison to nonadministered mice, anti-collagen antibodies in the sera of PG-administered mice did not alter. On the other hand, local expression of interleukin-17A (IL-17A), IL-6, IL-1**β**, interferon-**γ** (IFN-**γ**), C-C chemokine ligand 2 (CCL2), C-X-C chemokine ligand 1 (CXCL1), and CXCL2 in the joints of PG-administered mice decreased. Moreover, in the response of type II collagen- (CII-) restimulation ex vivo, IL-17A and IFN-**γ** production by splenocytes from PG-administered mice was less than that of control mice. These data suggested that daily ingested PG attenuated CIA pathogenesis by modulating immune response of splenocytes to CII stimulation and local production inflammatory cytokines and chemokines in the joints.

## 1. Introduction 

Rheumatoid arthritis (RA) is an autoimmune disease that is characterized by chronic inflammation of synovial joints, subsequently with progressive, erosive destruction of articular tissues [1]. It affects 1% of population and is associated with significant morbidity and mortality [2]. In the synovial tissues of RA, numerous cytokines are expressed and are functionally active. They are directly implicated in the immune processes that are thought to play crucial roles in the pathology of RA. In many rodent models, the cytokine modulation alters the outcome of arthritis [3].

Proteoglycans (PGs) are widely distributed in connecting tissues such as skin, bone, and cartilage by forming a complex with collagen, fibronectin, laminin, hyaluronic acid, and other glycoproteins [4–6]. Basic structure of PGs is a complex glycohydrate, which is composed of a core protein covalently attached with one or more glycosaminoglycan(s). Our previous studies have shown that PG extracted from salmon cartilage has the immunomodulatory effect. It suppresses inflammatory response of macrophages induced by stimulation with heat-killed bacteria [7]. In addition, daily oral administration of PG attenuates the severity of mouse experimental colitis and experimental autoimmune encephalomyelitis (EAE) [8, 9]. Attenuation of the systemic inflammation in colitis and EAE models by daily oral administration of PG depends on suppression of T-helper 17 (Th17) lineage differentiation and an induction of Foxp3^+^ regulatory T (Treg) cells [8, 9]. Our previous study also indicated that ingested PG may contribute to homeostasis of host immunity mediated through the balance in composition of gut microbial immunity [10].

In this study, the immunomodulatory effect of PG on the progression of arthritis was investigated. Mice with collagen-induced arthritis (CIA) were administered with PG per os daily. Our results demonstrated that immune response of splenocytes to collagen stimulation and proinflammatory cytokine and chemokine expression in the joints were modulated by oral administration of PG. These data suggested that PG has the prophylactic effect which is able to attenuate the severity of several inflammatory diseases not only colitis and EAE but also arthritis which is an important autoimmune disease.

## 2. Materials and Methods

### 2.1. Mice

DBA/1J mice were purchased from CLEA Japan, Inc., Tokyo, Japan. They were housed under specific-pathogen-free conditions in the Institute for Animal Experimentation, Hirosaki University Graduate School of Medicine. All animal experiments in this paper were conducted in accordance with the Animal Research Ethics Committee, Hirosaki University Graduate School of Medicine, and followed the Guidelines for Animal Experimentation, Hirosaki University.

### 2.2. Preparation and Administration of PG

Salmon cartilage PG was purchased from Kakuhiro Co., Ltd., Aomori, Japan. Lyophilized PG powder was dissolved in phosphate-buffered saline (PBS) given a concentration of 10 mg/mL. DBA/1J mice were administered with 2 mg of PG per os daily. PBS was used as control.

### 2.3. Induction of Arthritis

Arthritis was induced as described previously [11]. Briefly, 8- to 12-week-old female mice were immunized intradermally at the base of the tail with 50 *μ*g type II collagen (CII; MD Biosciences GmbH, Zürich, Switzerland) in complete Freund's adjuvant (CFA). To prepare the CFA, the desiccated killed* Mycobacterium tuberculosis* H37RA (BD Diagnostic Systems, Sparks, MD) was ground with a pestle and mortar and then suspended in incomplete Freund's Adjuvant (IFA, Sigma-Aldrich Co., Tokyo, Japan) to give a concentration of 4 mg/mL. To prepare CII in CFA, CII was dissolved in 10 mM acetic acid given a concentration of 4 mg/mL and emulsified in an equal volume of CFA. Mice were given a subcutaneous booster immunization with 50 *μ*g of CII in the IFA on day 21 after primary immunization.

### 2.4. Scoring for Evaluating Arthritis Severity in CIA

Mice were monitored for arthritis daily from day 18 after immunization. Each paw was scored for clinical signs of arthritis as follows: 0: no evidence of erythema and swelling, 1: erythema and swelling confined to digits, 2: erythema and mild swelling extending from ankle to tarsals, 3: erythema and moderate swelling extending from ankle to metatarsal joints, and 4: erythema and severe swelling encompass ankle, foot, and digits [11].

### 2.5. Histological Analysis

After completing the experiment, mice were sacrificed and paws were fixed in 10% neutral-buffered formalin and decalcified with 10% EDTA, pH 7.4. The paws were then embedded in paraffin and cut into 5-*μ*m thick sections. The deparaffinized sections were stained with hematoxylin and eosin and observed under light microscope. For immunostaining, the sections were incubated for 1 h at room temperature (RT) with monoclonal antibody (mAb) to mouse YL-6G/-6C neutrophil marker (1 : 50, Hycult Biotech, Uden, The Netherlands) or mAb to rat anti-mouse F4/80 antigen (1 : 100, AbD Serotec, Co., Oxford, UK). Goat anti-rat IgG2b conjugated with horseradish peroxidase (HRP, 1 : 250, AbD Serotec, Co.) was used, and immunoreactive cells were visualized by staining with 3,3-diaminobenzidine solution. Images were observed using virtual slide system (Olympus, Co., Tokyo, Japan).

### 2.6. Determination of Anti-CII Antibodies

Anti-CII antibodies in sera from mice were measured by enzyme-linked immunosorbent assays (ELISAs). Immunosorbent plates were coated with CII in carbonate buffer, pH 9.6 at 4°C for 16 h. After blocking with 10% Block Ace (BA, DS Pharma Biomedical, Osaka, Japan) in PBS for 1 h, sera (100 *μ*L) serially diluted with 10% BA in PBS were added into the plate and incubated for 2 h at RT. After washing, HRP-conjugated goat anti-mouse IgG1 or IgG2a was added into the plate and incubated for 2 h at RT. Then, the mixture of* o*-phenylenediamine and H_2_O_2_ in citrate phosphate buffer was added to each well. The color reaction was stopped with 8 N H_2_SO_4_. The absorbance was measured at 490 nm with reference of 655 nm wavelength using microplate reader model 680 (Bio-Rad, Tokyo, Japan).

### 2.7. Quantitative Real-Time PCR

The paws were collected during days 20–30 after booster immunization and mechanically homogenized. Total RNA was prepared from samples using TRIzol reagent (Invitrogen Co., Carlsbad, CA) according to manufacturer's instruction. First-strand cDNA was synthesized by reverse transcription of 1 *μ*g total RNA using random primers (Takara Shuzo, Kyoto, Japan) and Moloney murine leukemia virus reverse transcriptase (Invitrogen). SYBR Green Supermix (Bio-Rad Laboratories, Hercules, CA) was used for quantitative real-time PCR. Primers used in this study were described in Table 1. PCRs were run at the following conditions: initial activation of Taq DNA polymerase at 95°C for 5 min, 40 cycles of 30 sec at 95°C for denaturing, 30 sec at 60°C for annealing, and 30 sec at 72°C for elongation. The detection threshold was set to the log linear range of the amplification curve and kept constant (0.05) for all data analysis. Threshold cycle (*C*_*T*_) of each target product was determined and set in relation to the amplification plot of glyceraldehyde-3-phosphate dehydrogenase (GAPDH). Difference in *C*_*T*_ values (Δ*C*_*T*_) of two genes was used to calculate the relative expression (relative expression = 2^−(*C*_*T*_  of  target  genes−*C*_*T*_  of  GAPDH)^ = 2^−Δ*C*_*T*_^).

### 2.8. Ex Vivo Cytokine Production of CII Restimulated Splenocytes

T-cell proliferative responses to CII were measured with standard microtiter assay using splenocytes from mice immunized with CII [11]. Mice were sacrificed on day 10 after primary immunization. The spleen was collected and single-cell suspension was prepared. Red blood cells in suspension were lysed with 0.83% ammonium chloride. Splenocytes were cultured in Dulbecco's modified Eagle medium (DMEM, Nissui Pharmaceutical Co., Tokyo, Japan) supplemented with 10% fetal calf serum (JRH Biosciences, Lenexa, KS) and 0.03% L-glutamine (Wako Pure Chemical Industries, Osaka, Japan) at 5 × 10^6^ cells/mL in 24-well plates. Splenocytes in DMEM were restimulated with CII 50 *μ*g/mL. The supernatants were collected 48 h later. Determination of interferon-*γ* (IFN-*γ*) production was carried out by ELISAs as described previously [12]. Interleukin-17A (IL-17A) titers were determined by mouse IL-17A platinum ELISA (Bender MedSystems GmbH, Vienna, Austria) according to manufacturer's instruction.

### 2.9. Statistical Analysis

Statistical significance was determined by a two-tailed, unpaired Student's* t*-test. Differences in arthritis incidence were analyzed using Fisher's exact test. *P* values lower than 0.05 are considered to be significant.

## 3. Results

### 3.1. Attenuation of CIA Severity by Daily Oral Administration of PG

**Figure 1:**
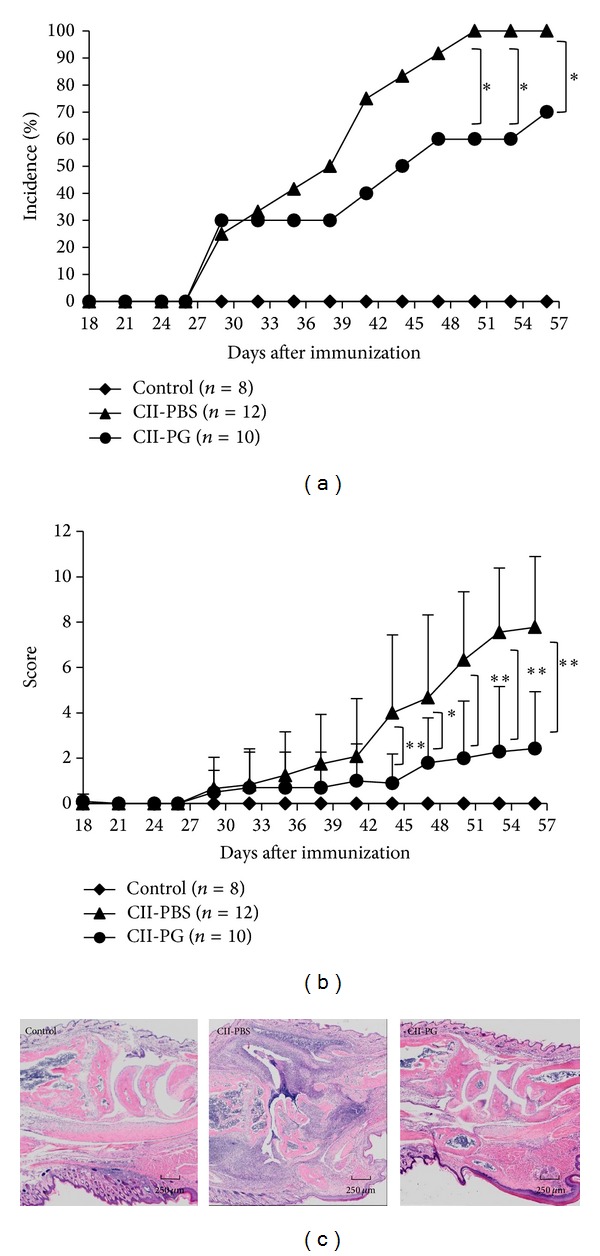
Oral administration of PG attenuated severity of arthritis in CII-immunized mice. (a) Percent incidence of arthritis, (b) clinical scores of arthritis, and (c) HE staining of ankle joints. Mice were immunized with CII as described in Section 2. From the day of the first CII immunization, mice were orally administered with PG (2 mg per mouse) once a day. Incidence and clinical scores of CIA were recorded between days 18 and 56. On day 56 of the first CII-immunization, joints were collected for histological analysis. Control, CII-PBS, and CII-PG indicate nonimmunized mice, CII-immunized mice administered with PBS, and CII-immunized mice administered with PG, respectively. Data are representative of 3 independent experiments (*n* = 2–4 mice per group). Single asterisk (*P* < 0.05) and double asterisk (*P* < 0.01) indicate significant difference between PG- and PBS-administered groups.

In order to investigate the effect of PG on CIA, CII-immunized mice were administered with 2 mg of PG per os daily by starting on the day of the first CII immunization. Clinical scores of CIA were recorded between day 18 and day 56 after the first immunization. Percent incidence and clinical scores of CIA in the PG-administered mice decreased in comparison with PBS-administered mice (Figures 1(a) and 1(b)). From day 45 after the first immunization, the average clinical scores of CIA in the PG-administered mice were significantly different from that of PBS-administered mice (*P* < 0.05). In comparison with PBS-administered mice, histological analysis of joints showed that synovitis and osteoclastic bone resorption were attenuated by daily oral administration of PG (Figure 1(c)).

### 3.2. Decrease in Macrophages, Neutrophils, and Osteoclasts in the Joint of PG-Administered Mice

**Figure 2:**
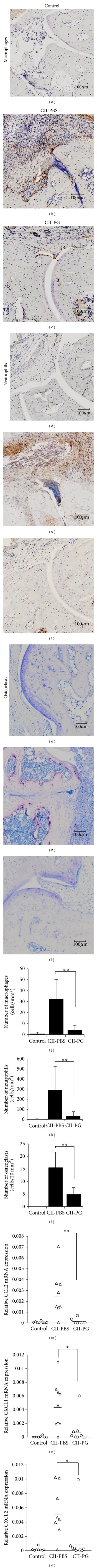
Administration of PG-attenuated infiltration of macrophages and neutrophils, accumulation of osteoclasts, and chemokine expression in joints of CII-immunized mice. ((a)–(c)) Immunostaining of macrophages, ((d)–(f)) immunostaining of neutrophils, ((g)–(i)) TRAP staining for osteoclasts, ((j)–(l)) quantitative analysis of macrophages, neutrophils, and osteoclasts, and ((m)–(o)) relative mRNA expression of CCL2, CXCL1, and CXCL2 in the joints of CII-immunized mice. Control, CII-PBS, and CII-PG indicate nonimmunized mice, CII-immunized mice administered with PBS, and CII-immunized mice administered with PG, respectively. Single asterisk (*P* < 0.05) and double asterisks (*P* < 0.01) indicate that the value is significantly different.

Infiltration of inflammatory cells and accumulation of osteoclasts play the important role in the progression of arthritis. To observe these cells in the joints of CII-immunized mice, immunostaining using markers for macrophages and neutrophils and TRAP staining for osteoclasts were performed. Infiltration of macrophages (Figures 2(a)–2(c) and 2(j)) and neutrophils (Figures 2(d)–2(f) and 2(k)) and accumulation of the osteoclasts (Figures 2(g)–2(i) and 2(l)) decreased in the joints of PG-administered mice. These results suggested that administration of PG suppresses infiltration of inflammatory cells and accumulation of osteoclasts in the joints of CII-immunized mice.

### 3.3. Reduction of Chemokine Expression in the Joints of PG-Administered Mice

Osteoclast activation and infiltration of inflammatory cells are regulated by chemokines. Thus, we then examined the chemokine production in the ankle joints of CII-immunized mice. Between day 39 and day 47 in which all CII-immunized mice exhibited clinical scores, total cellular RNA from ankle joints was extracted. The expression of C-C chemokine ligand 2 (CCL2), C-X-C chemokine ligand 1 (CXCL1), and CXCL2 in the paws of CII-immunized mice was determined by quantitative real-time PCR. Their expression in the joints of PG-administered mice significantly decreased, comparing with PBS-administered mice (Figures 2(m)–2(o)). The results correlated with the amounts of inflammatory cells observed in Figures 2(a)–2(l).

### 3.4. PG Administration Reduced Cytokine Expression in the Joints of CII-Immunized Mice

**Figure 3:**
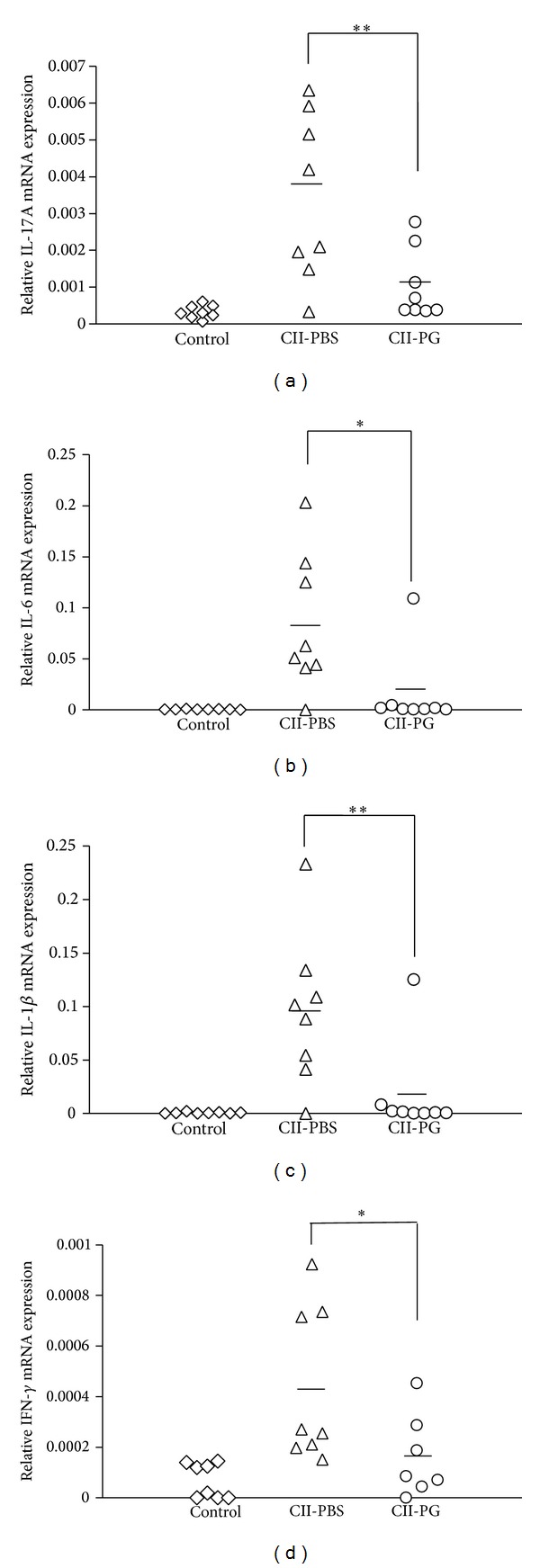
Administration of PG reduced cytokine expression in joints of CII-immunized mice. The expression of IL-17A (a), IL-6 (b), IL-1*β* (c), and IFN-*γ* (d) in joints was determined by quantitative real-time RT-PCR. Control, CII-PBS, and CII-PG indicate nonimmunized mice, CII-immunized mice administered with PBS, and CII-immunized mice administered with PG, respectively. Single asterisk (*P* < 0.05) and double asterisk (*P* < 0.01) indicate that the value is significantly different.

Proinflammatory cytokines play important roles in severity of RA. IL-17 triggers recruitment of macrophages and neutrophils through induction of chemokines [13, 14] and also upregulates other proinflammatory cytokines, such as IL-6 and IL-1*β*. Therefore, we investigated whether PG administration affects the expression of these proinflammatory cytokines. On days 39–47 after the first immunization, the levels of these cytokines were determined by quantitative real-time PCR. The expression of IL-17A, IL-6, IL-1*β*, and IFN-*γ* in the joints of PG-administered mice was reduced in comparison with PBS-administered group (Figures 3(a)–3(d)). These results suggested that PG administration modulates the expression of proinflammatory cytokines in the joint of CII-immunized mice at the late stage of RA.

### 3.5. Administration of PG Suppressed the Cytokine Production from Splenocytes in CII Restimulation

**Figure 4:**
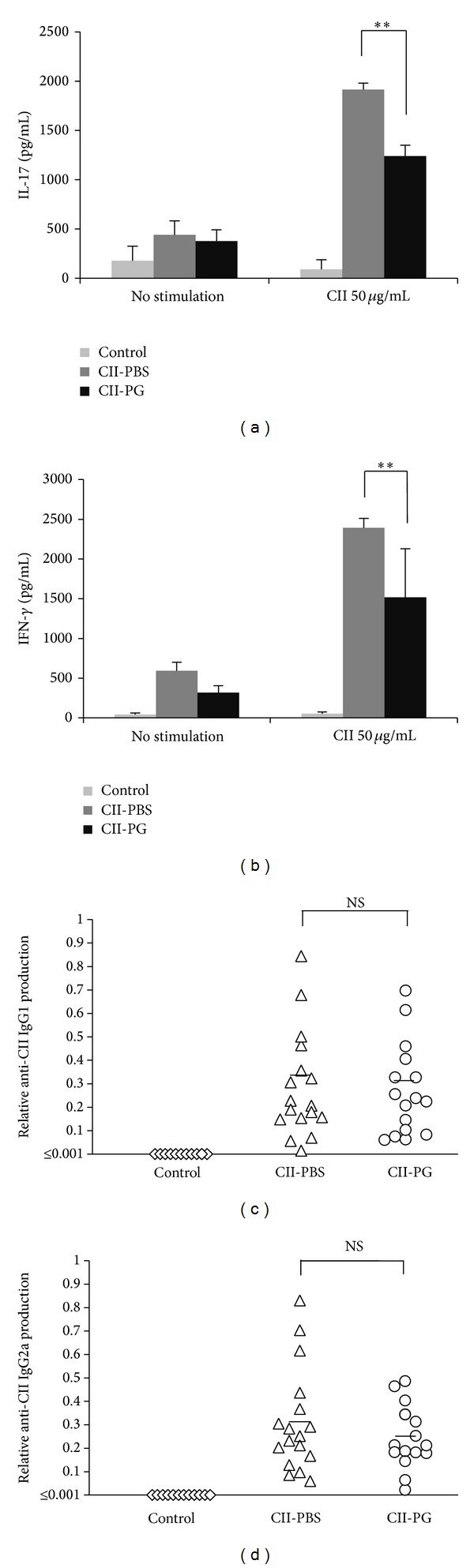
Administration of PG suppressed CII-specific cytokine response of splenocytes but did not alter CII-specific antibody production in serum. ((a)-(b)) CII-specific cytokine response of splenocytes. On day 10 of the first CII-immunization, splenocytes were collected and stimulated with CII ex vivo for 48 h. Titers of IL-17A (a) and IFN-*γ* (b) were determined from culture supernatants by ELISAs. The results are expressed as the means ± SD of 3 independent experiments. ((c)-(d)) CII-specific antibody production in serum. Sera of CIA mice were collected at the end of experiments. Anti-CII IgG1 (c) and IgG2a (d) levels in serum were determined by ELISA. Control, CII-PBS, and CII-PG indicate nonimmunized mice, CII-immunized mice administered with PBS, and CII-immunized mice administered with PG, respectively. Double asterisk (*P* < 0.01) indicates that the value is significantly different. NS: not significantly different.

The immune response to CII in CIA is characterized by the stimulation of collagen-specific T cells [11]. We examined the response of splenocytes to restimulation with CII ex vivo. CII-stimulated splenocytes from PBS-administered mice released high titers of IL-17 and IFN-*γ*, while administration of PG significantly suppressed both cytokines from CII-stimulated splenocytes (Figures 4(a) and 4(b)). These data suggested that administration of PG suppresses the T-cell response to restimulation with CII in splenocytes ex vivo.

### 3.6. PG Administration Did Not Affect Antibody Responses

Anti-CII antibodies are reportedly involved in pathology of arthritis in CII-immunized mice [15]. Therefore, we determined the production of CII-specific immunoglobulin isotypes, IgG1, and IgG2a, in these mice by ELISAs. The production of anti-CII specific IgG1 or IgG2a antibody in the sera increased upon CII-immunization and was not significantly different between PBS- and PG-administered groups (Figures 4(c) and 4(d)). These results suggested that attenuation of CIA severity by oral administration of PG is not involved in the modulation of anti-CII antibody production.

## 4. Discussion

To assess the efficacy of immunomodulatory agents for RA therapy in humans, CIA is a promising experimental model [15]. Chronic inflammation of joints is a main characteristic of RA and CIA [1, 16]. It has been reported that autoantibodies to CII and CII-specific T-cell response play an important role in the pathogenesis of CIA [11]. Synovitis of RA is characterized by infiltration of inflammatory cells and chemokine production [1]. In CIA, monocyte migration into joint associates with CCL2 [17] and CXCL1 and CXCL2 derived from macrophages act as neutrophil chemoattractants [18, 19]. Inflammatory cells migrated into the joints produce several proinflammatory cytokines including tumor necrosis factor-*α* (TNF-*α*), IL-6, and IL-1 [3, 14, 20]. Moreover, IL-17 cytokine family, particularly IL-17A, has been implicated in the pathogenesis of RA in humans [21]. This cytokine upregulates receptor activator of nuclear factor-*κ*B ligand (RANKL) on synovial fibroblasts and triggers local inflammation [13, 22]. IL-17A activates synovial macrophages to produce proinflammatory cytokines such as IL-6 and IL-1 [3, 13]. IL-6 and IL-1 induce osteoclastogenesis by either acting directly on osteoclast precursor cells or upregulates RANKL on synovial fibroblasts [13]. Osteoclasts thus activated by proinflammatory cytokines are thought to be responsible for bone erosion [3, 20].

We presumed that salmon PG displays a potential to reduce the pathogenesis of RA because of its immunomodulating activities [7–9]. The early stage of CII-immunization is considered as an important time point for immunopathogenesis of CIA. Thus, daily administration of PG was initiated on the same day of the first CII-immunization. Our results demonstrated that severity of CIA was attenuated by PG administration. We also found that PG modulated CII-specific cytokine responses but not CII-specific IgG1 and IgG2a production. These results implied that PG might not affect humoral-mediated immune response. This finding correlates to our results obtained from the allergic mouse models. Oral administration of PG showed no influence on antigen-specific antibodies in ovalbumin- and peanut-induced allergic mice (unpublished observations). PG is a main extracellular matrix component in joint and can act as an autoantigen for generating autoantibody. In fact, PG from mice has been shown to induce arthritis in animal models [23, 24]. However, our results demonstrated that neither antibodies to PG nor arthritis was observed by daily oral administration of PG (data not shown). It is presumed that this may be due to a different route of administration and salmon PG may not induce autoantibodies in the condition employed herein.

In addition to macroscopic evaluation, the effect of PG on severity of CIA was investigated by histopathological analysis. In the joints of PG-administered mice, accumulation of osteoclasts was significantly reduced as well as infiltration of macrophages and neutrophils. This result correlates to the expression of CCL2, CXCL1, and CXCL2 chemokines. In CIA, inflammatory cytokines in the joints play crucial roles in the pathology [14, 25, 26]. We observed that the expression of IL-17A, IL-6, IL-1*β*, and IFN-*γ* in the joints of PG-administered mice was significantly reduced. The role of IFN-*γ* in animal models of arthritis is complex. In the early stage of CIA, IFN-*γ* inhibits Th17 differentiation and thereby reduces inflammation [27]. In contrast, the committed Th-17 cells that have already existed at the late stage of CIA resist inhibition by IFN-*γ*. Alternatively, IFN-*γ* at the late stage of CIA has been reportedly shown to induce inflammation [28]. Thus, IFN-*γ* produced at the late stage of CIA in our experiments might be involved in severity of synovitis.

Our previous studies demonstrated that PG reduces TNF-*α* production and induces Foxp3^+^ Treg cells in colitis and EAE mouse models [8, 9]. However, in this CIA model, TNF-*α* and Foxp3 expression in the joints was not significantly altered by PG administration (data not shown). Although the reason of these results remained unclear, overall of our data indicated that daily administration of salmon PG attenuates inflammation and severity in CIA. This finding implied that salmon PG is a promising prophylactic agent. Continuous consumption of PG from salmon might be able to reduce the progression of inflammatory and autoimmune diseases.

## Figures and Tables

**Table 1:** Primers and PCR conditions used in this study.

Product	Primer sequence (5′-3′)	
	Forward	Reverse
GAPDH	TGAAGGTCGGTGTGAACGGATTTGG	ACGACATACTCAGCACCGGCCTCAC
CCL2	ACTGAAGCCAGCTCTCTCTTCCTC	TTCCTTCTTGGGGTCAGCACAGAC
CXCL1	GGATTCACCTCAAGAACATCCAGAG	CACCCTTCTACTAGCACAGTGGTTG
CXCL2	GAACAAAGGCAAGGCTAACTGA	AACATAACAACATCTGGGCAAT
IL-17A	CCTCAAAGCTCAGCGTGTCC	GAGCTCACTTTTGCGCCAAG
TNF-*α*	GGCAGGTCTACTTTGGAGTCATTGC	ACATTCGAGGCTCCAGTGAATTCGG
IL-6	TGGAGTCACAGAAGGAGTGGCTAAG	TCTGACCACAGTGAGGAATGTCCAC
IL-1*β*	AAGGAGAACCAAGCAACGACAAAA	TGGGGAACTCTGCAGACTCAAACT
IFN-*γ*	AGCGGCTGACTGAACTCAGATTGTAG	GTCACAGTTTTCAGCTGTATAGGG
